# Feasibility Study of Nose-to-Brain Delivery of Galantamine for Alzheimer’s Disease: Enhancing Olfactory-Region Deposition to Improve Therapeutic Efficacy

**DOI:** 10.3390/pharmaceutics18070885

**Published:** 2026-07-20

**Authors:** Chuangxin Chen, Chunying Leung, Zizhao Zhai, Guanlin Wang, Rui Yang, Qiuyi Hu, Xiao Yue, Zhongxuan Yao, Ziyu Zhao, Xuejuan Zhang

**Affiliations:** 1 State Key Laboratory of Bioactive Molecules and Druggability Assessment, Guangdong Basic Research Center of Excellence for Natural Bioactive Molecules and Discovery of Innovative Drugs, Jinan University, Guangzhou 510006, China; c.x.chen@foxmail.com (C.C.); lcy0301@stu2021.jnu.edu.cn (C.L.); zhaizizhao@stu2021.jnu.edu.cn (Z.Z.); ruiyang@stu2023.jnu.edu.cn (R.Y.); huqiuyi928@163.com (Q.H.); yuex_1998@163.com (X.Y.); runablebox@stu2021.jnu.edu.cn (Z.Y.); zhaoziyu0515@163.com (Z.Z.); 2 College of Pharmacy, Jinan University, Guangzhou 510006, China; 3 School of Pharmaceutical Sciences, Sun Yat-sen University, Guangzhou 510006, China; wangglin3@mail2.sysu.edu.cn

**Keywords:** nose-to-brain drug delivery, Alzheimer’s disease, galantamine, olfactory deposition, spray performance

## Abstract

**Background**: Alzheimer’s disease (AD) is the seventh leading cause of death worldwide, posing a substantial global health burden. Although galantamine (GNT) is a first-line clinical drug for AD treatment, its therapeutic efficacy is constrained by inefficient brain delivery across the blood–brain barrier (BBB). Nose-to-brain delivery represents a promising route to bypass the BBB. However, its efficiency remains limited by insufficient drug deposition in the anatomically restricted olfactory region. In this study, we developed a galantamine nasal spray (GNT-NS) with enhanced olfactory region deposition and evaluated its feasibility for nose-to-brain delivery in AD treatment. **Methods**: We optimized the formulation by systematically investigating the cascade relationship among formulation physicochemical properties, spray performance, and olfactory region deposition. Nasal deposition distribution was quantitatively evaluated using a physiologically realistic 3D-printed human nasal cavity model reconstructed from clinical magnetic resonance imaging (MRI) data. Further, the *in vivo* biodistribution and therapeutic efficacy of GNT-NS were evaluated in AD rats. **Results**: The optimized formulation P_3_ achieved an olfactory region fraction of 23.85%, markedly exceeding that of the unoptimized formulation P_0_. Subsequent *in vivo* biodistribution studies showed that P_3_ produced higher brain drug exposure than both intranasally administered P_0_ and the commercial oral formulation. Further pharmacodynamic studies demonstrated that GNT-NS significantly improved cognitive and behavioral deficits in AD rats, exhibiting superior therapeutic efficacy over commercially available oral galantamine tablets. **Conclusions**: Collectively, this study proposes a cascade regulation strategy linking formulation physicochemical properties, spray performance, and olfactory region deposition and demonstrates that optimizing nasal spray properties can enhance olfactory deposition, increase brain exposure and improve therapeutic efficacy. These findings provide a useful reference for the design of nose-to-brain delivery formulations for AD and other central nervous system diseases.

## 1. Introduction

Alzheimer’s disease (AD) is a prevalent and progressive neurodegenerative disorder, ranking as the seventh leading cause of death worldwide [1]. Currently, AD affects over 55 million people globally, causing economic losses exceeding 305 billion dollars annually and placing a substantial burden on public health systems [1,2,3,4,5]. AD is primarily characterized by a progressive deficit in cholinergic neurotransmission. Acetylcholinesterase inhibitors (AChEIs) are approved as first-line treatments for AD, restoring cholinergic signaling by inhibiting acetylcholine (ACh) hydrolysis [6]. Among these, galantamine (GNT) possesses a unique dual mechanism of action: it acts both as an acetylcholinesterase inhibitor to elevate synaptic acetylcholine levels and as an allosteric modulator of nicotinic acetylcholine receptors (nAChRs) to enhance their responsiveness. Therefore, GNT exhibits broader neuroprotective and cognitive-enhancing effects than conventional AChEIs that act through cholinesterase inhibition alone [4].

Currently, commercial formulations of GNT are primarily oral tablets and injections. However, their therapeutic efficacy is suboptimal, as the blood–brain barrier (BBB) severely restricts the transport of systemically administered GNT into the brain parenchyma [7,8]. Pharmacokinetic data from relevant studies indicate that GNT exhibits poor BBB permeability, with its brain tissue exposure reaching only 12.5% of that in plasma [9]. This creates a clinical dilemma: achieving effective therapeutic concentrations in the brain requires high systemic doses, which inevitably lead to excessive peripheral drug accumulation and severe adverse events, particularly gastrointestinal distress [10,11]. Therefore, there is an urgent need to develop strategies that enhance the brain delivery of GNT.

In this context, nose-to-brain drug delivery has emerged as a promising non-invasive strategy. Upon intranasal administration, drugs can bypass the BBB and be transported directly to the brain via the olfactory and trigeminal nerve pathways [12]. Compared with oral administration, this approach offers multiple advantages, including high brain bioavailability, rapid onset of action, and the avoidance of first-pass metabolism [13,14,15]. However, actual brain delivery efficiency remains limited by multiple factors, including the olfactory region fraction (ORF), mucociliary clearance, epithelial permeability, nasal residence time, and perineural transport mechanisms. The olfactory region, containing a high density of olfactory nerve fibers, serves as the primary portal for nose-to-brain delivery [16,17]. Nevertheless, the anatomically narrow nasal valve region severely hinders drug delivery to the deep olfactory area [18,19]. According to previous reports and our preliminary experiments, the ORF of traditional commercial nasal sprays is typically only 1% to 5% [20,21,22]. Therefore, effective strategies must be developed to increase the ORF of GNT, thereby enhancing nose-to-brain delivery efficiency.

Studies have indicated that the spray performance of nasal sprays (including plume geometry, spray pattern, and droplet size distribution) is a critical factor governing drug deposition distribution within the nasal cavity. For instance, a narrower plume angle can facilitate droplet passage through the narrow nasal valve structure [5]. Crucially, spray performance is highly dependent on formulation properties [23]. Optimizing spray performance by modulating formulation characteristics presents a simpler, cost-effective, and easily industrialize strategy. Our previous research has demonstrated that altering the rheological properties of nasal spray can significantly affect spray droplet behavior, thereby effectively regulating the nasal deposition distribution of drugs [16]. However, current research on nose-to-brain delivery has mainly focused on post-deposition absorption enhancement, such as nanocarrier design. Less attention has been paid to improving initial drug delivery to the olfactory region. Therefore, increasing the ORF through simple formulation modulation may help address a key efficiency bottleneck in nose-to-brain delivery and support the development of more translatable intranasal formulations for AD.

**Figure 1:**
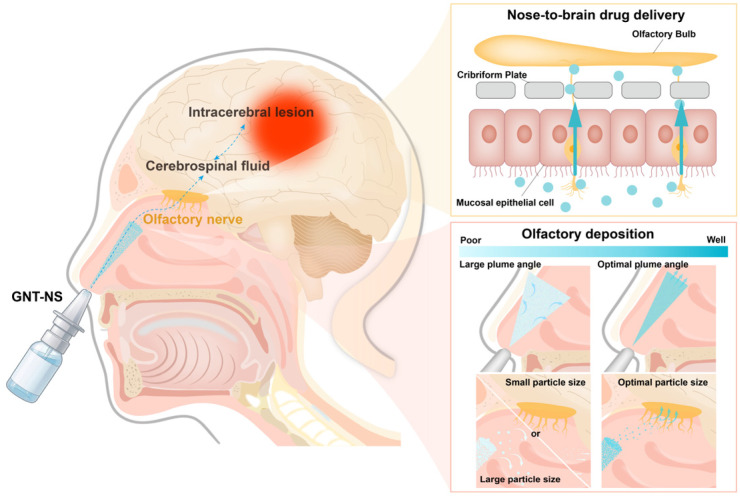
Tailored olfactory-region deposition and pharmacodynamic efficacy of GNT-NS for AD treatment. The arrows indicate the deposition of spray droplets in the olfactory region and their subsequent absorption and transport along the nose-to-brain pathway.

Herein, we developed a galantamine nasal spray (GNT-NS) with enhanced olfactory deposition by modulating formulation viscosity and evaluated its feasibility for nose-to-brain delivery in the treatment of Alzheimer’s disease (Figure 1). The effects of formulation physicochemical properties on plume geometry, spray pattern, and droplet size distribution of GNT-NS were systematically investigated. Drug distribution within the nasal cavity was quantitatively evaluated using a 3D-printed human nasal cavity model, thereby to investigate the cascade relationship among physicochemical properties, spray performance, and olfactory deposition. Subsequently, the in vivo brain biodistribution and pharmacodynamic efficacy of the optimized GNT-NS were evaluated. This study suggests that rational optimization of nasal spray physicochemical properties can enhance olfactory-region deposition, and increase brain drug exposure, thereby potentially improving the pharmacodynamic efficacy of galantamine for AD treatment via the nose-to-brain route.

## 2. Materials and Methods

### 2.1. Chemicals

Galanthamine hydrobromide, sodium chloride (NaCl), ethylene diamine tetra acetic disodium (EDTA-2Na), normal saline solution, disodium hydrogen phosphate (NaH_2_PO_4_) and polyvinylpyrrolidone K90 (PVP) were obtained from Shanghai Yuanye Bio-Technology Co., Ltd. (Shanghai, China). The commercially available galantamine hydrobromide tablets (JinKang^®^, 5 mg/tablet) were obtained from CONBA Pharmaceutical Co., Ltd. (Zhejiang, China). Dodecyl-*β*-D-Maltoside was sourced from Meryer Chemical Technology Co., Ltd. (Shanghai, China). Methanol was purchased from Thermo Fisher (Boston, MA, USA). Triethylamine was supplied by Kemiou Chemical Reagent Co., Ltd. (Tianjin, China). Benzalkonium Chloride was acquired from Taiko Palm-Oleo Co. Ltd. (Zhangjiagang, China). The beta-amyloid protein fragment 1–42 (A*β*_1–42_) and phosphoric acid were procured from Shanghai Macklin Biochemical Technology Co., Ltd. (Shanghai, China).

### 2.2. Animal and Cell

Adult male Sprague-Dawley (SD) rats, weighing 220–250 g and classified as specific pathogen-free (SPF), were obtained from the Laboratory Animal Center at Sun Yat-sen University in Guangzhou, China. These rats were kept in a controlled setting where the temperature was regulated between 20 and 25 °C and humidity was maintained at 50 to 60%. The rats were acclimatized to the laboratory environment for 3 days prior to the experiments. All animal experiments were conducted following the guidelines of laboratory animals supervised by the Institutional Animal Care and Use Committee of Sun Yat-sen University (Approval Number: SYSU-IACUC-2026-B2174). Additionally, the Human Lung Adenocarcinoma Epithelial Cells (A549) and RPMI 2650 cells used in this research were kindly supplied by the School of Pharmaceutical Sciences at Sun Yat-sen University.

### 2.3. Preparation and Characterization of GNT-NS

The GNT-NS formulations were prepared sequentially. First, EDTA-2Na (0.5% *w*/*v*) and NaH_2_PO_4_ (0.1% *w*/*v*) were dissolved in ultrapure water, followed by the addition of the absorption enhancer DDM (0.25% *w*/*v*) and the preservative BAK (0.01% *w*/*v*). Subsequently, GNT hydrobromide (25 mg/mL) and sodium chloride (0.6% *w*/*v*, for osmotic adjustment) were dissolved into the mixture. To prepare formulations with varying viscosities (P_0_~P_5_), the mixture was aliquoted, and graded concentrations of PVP were added. The solutions were allowed to equilibrate for 5 h to ensure complete polymer hydration and homogeneity. Finally, the formulations were filtered through a 0.22 μm microporous membrane and filled into VP7 spray devices (Aptar Pharma, Suzhou, China). The detailed compositions of P_0_~P_5_ are listed in Table 1.

The viscosity, osmotic pressure, surface tension, and pH of the GNT-NS solutions were determined using a DVNext^®^ viscometer (Brookfield, Halifax, NS, Canada), an osmometer (STY-1A, Tianjin Tianda Tianfa Technology Co., Ltd., Tianjin, China), a fully automatic surface tensiometer (BZY-1, Shanghai Hengping Instrument and Meter Factory, Shanghai, China), and a pH meter (Mettler-Toledo, Zurich, Switzerland), respectively. The single pump delivery and single actuation content of GNT-NS were quantified using a Shimadzu^®^ LC-20 high-performance liquid chromatography (HPLC) system (Shimadzu Co., Kyoto, Japan). Separation was achieved on an Agilent^®^ C18 column (250 mm × 4.6 mm i.d., 5 μm particle size; Agilent Technologies, Santa Clara, CA, USA) with UV detection at 289 nm. The mobile phase consisted of a mixture of ultrapure water/methanol (75:25, *v*/*v*) with 0.2% triethylamine (pH adjusted to 6.0 using phosphoric acid) and was delivered at a flow rate of 1.0 mL/min. Unless otherwise stated, all in vitro formulation-related experiments in this study were performed using three independently prepared samples or batches.

### 2.4. Droplet Size Distribution Performance

The droplet size distribution was evaluated using laser diffraction particle size analyzers (Sympatec HELOS & SPRAYER^TM^, Sympatec GmbH, Clausthal-Zellerfeld, Germany). The measurement parameters included an actuating force of 60 N, an R4 lens, and a 300 ms recording duration. The spraying process was divided into three phases: formation, stabilization, and dissipation. Data acquisition was triggered and terminated at an optical concentration (*C*_opt_) threshold of 0.5%. The results were characterized by *D*_10_, *D*_50_, and *D*_90_, representing the 10%, 50%, and 90% percentiles of the cumulative droplet size distribution, respectively. All samples were analyzed in triplicate.

### 2.5. Plume Geometry and Spray Pattern Evaluation

The plume’s geometry and spray pattern were assessed using a SprayVIEW^®^ measurement system (Proveris Scientific Corporation, Hudson, MA, USA). The GNT-NS devices were actuated by a velocity-controlled Vereo Automated Actuator System (Proveris Inc.) with an activation acceleration of 2500 mm/s^2^ and a hold time of 100 ms. The plume angle, plume distance, spray area, and ellipticity were measured at distances of 30 mm and 60 mm.

### 2.6. In Vitro Nasal Distribution and Deposition of GNT-NS

A 3D-printed nasal cast based on a previously reported adult MRI-derived nasal cavity structure was used as a human nasal surrogate to evaluate the drug deposition profile [23]. The nasal cast, printed with RESIN-A and encased in an airtight plastic shell, comprised five anatomical regions: the nasal vestibule (NV), inferior nasal tract (INT), middle nasal tract (MNT), superior nasal tract (SNT), and oropharynx (ORO). The setup was connected to an impactor (Copley Controls Corp., Akron, OH, USA) operating at a continuous inhalation flow rate of 15 ± 0.5 L/min.

GNT-NS formulations were delivered into the right nostril of the cast using VP7 devices. The nozzles were inserted to a depth of 5 mm, at a 60° angle to the horizontal and a 0° angle to the coronal plane [24]. Each trial consisted of four activations (100 μL per spray). Immediately after administration, the cast was disassembled to avoid droplet redistribution. The NV, INT, MNT, SNT, and ORO sections were rinsed with ultrapure water, and the deposited GNT content in each segment was quantified using HPLC.

### 2.7. Biodistribution Study of GNT-NS

An IVIS Lumina Series III small-animal imaging system (PerkinElmer, Waltham, MA, USA) was used to evaluate the in vivo biodistribution of GNT-NS and commercially available oral galantamine tablets after administration. Rhodamine B was incorporated into the formulations as a fluorescence tracer. SD rats were anesthetized with isoflurane. Rhodamine B-labeled GNT-NS was administered by intranasal spray at a volume of 100 μL per rat, while Rhodamine B-labeled oral galantamine solution was administered by oral gavage at an equivalent dose. At 2 h post-administration, the brain tissue, brain slicing, nasal cavity, trigeminal nerves, heart, liver, spleen, lung, and kidney were collected for ex vivo fluorescence imaging. Fluorescence images were acquired and analyzed using Living Image software (version 4.4; Caliper Life Sciences, Hopkinton, MA, USA). To ensure consistency among groups, all images were processed with identical scale and background-subtraction settings. After background subtraction, total radiant efficiency was used to express the relative fluorescence signal in each region.

### 2.8. In Vivo Pharmacodynamic Studies of GNT-NS on AD Rats

#### 2.8.1. Model Establishment of AD Rats and Administration Scheme

The AD rat models were induced by injecting A*β*_1–42_ into the hippocampus. Using the bregma as the stereotaxic reference point, the injection coordinates were set according to the rat brain atlas: A/P −3.0 mm, L/R ±3.5 mm, and depth 4.0 mm [18]. A total of 5 μL of A*β*_1–42_ (1 μg/μL) was microinjected into the CA1 region at a rate of 1 μL/min. The micro-syringe was left in place for 5 min post-injection to prevent backflow.

The stereotaxic procedure was performed under anesthesia. Animals were monitored daily, and humane endpoints included failed recovery, severe distress, marked weight loss, or inability to eat or drink. The sample size was determined based on previous AD animal studies with comparable behavioral endpoints and the 3R principle, with six rats included per group [25,26,27,28]. Before randomization, animals without obvious health or behavioral abnormalities after acclimatization were included. Animals with severe procedure-related complications, failed recovery, or conditions interfering with behavioral assessment were excluded a priori. Data points were excluded only when technical failure prevented reliable analysis. No animals, experimental units, or data points were excluded from the final analysis.

After model establishment, the experimental rats were randomly allocated into four groups using a computer-generated random number sequence: healthy control, AD model, commercial galantamine oral formulation (GNT-PO), and GNT-NS intranasal groups. The GNT-PO and GNT-NS groups received galantamine hydrobromide equivalents at 1.8 mg/kg/day for 4 weeks. This dose was determined by body-surface-area conversion from the clinically used adult dose of galantamine tablets, following standard interspecies dose-conversion principles. All intranasal administrations were performed at a volume of 100 μL using an HRH-MAG4 metered liquid atomizer (Beijing Huironghe Technology Co., Ltd., Beijing, China). The healthy control and AD model groups received sterile physiological saline once daily using the same procedure.

Randomization and drug administration were performed by an investigator not involved in behavioral testing, histological evaluation, image quantification, or statistical analysis. Treatment and testing orders were balanced across groups, and all animals were housed and tested under the same environmental conditions. Outcome assessors and data analysts were blinded to group allocation until the analyses were completed.

#### 2.8.2. Learning and Memory Assessment Using the Morris Water Mazes

The standard Morris water maze test conducted to evaluate the learning and memory abilities of AD rats. A dark circular pool (1.6 m in diameter, 1.0 m in depth) was filled with water, and a digital camera (HY1080, Jierruiweitong, Shenzhen, China) was mounted above to track the rats’ swimming paths. An escape platform (10 cm in diameter) was submerged 2 cm below the water surface in the target quadrant. Rats were randomly released from four starting quadrants to locate the hidden platform during four consecutive days of training. During the 60 s observation window, key parameters including escape latency, the time proportion on the target quadrant, and swimming speed were recorded. Escape latency in the Morris water maze test was predefined as the primary outcome measure for evaluating cognitive improvement and was used as the main behavioral endpoint for sample size determination.

#### 2.8.3. Exploration Behavior and Anxiety Assessments Using the Elevated Plus Maze

The elevated plus-maze test (XR-XG201, Shanghai Xinruan Information Technology Co., Ltd., Shanghai, China) was performed to assess exploratory behavior and anxiety in AD rats. The apparatus consisted of a central platform radiating two open and two enclosed arms. Each rat was placed on the central platform facing an open arm, and its behavior was recorded for 5 min using a video tracking system equipped with ANY-maze Video Tracking System software (version 7.20, 64-bit; Stoelting Co., Wood Dale, IL, USA). The recorded parameters included the number of entries and the residence duration in both the open and closed arms.

#### 2.8.4. Exploration Behavior and Anxiety Assessments Using the Open-Field Test

The open-field test (XR-XZ301, Shanghai Xinruan Information Technology Co., Ltd., Shanghai, China) was used to assess the spontaneous exploratory behavior and anxiety of AD rats. Each rat was placed in a 50 cm × 50 cm × 50 cm (length × width × height) black Plexiglas box in a quiet environment. An overhead camera connected to ANY-maze Video Tracking System software recorded the locomotor paths for 5 min. Recorded parameters included the total distance traveled, average speed, movement duration, and the number of entries into different zones.

#### 2.8.5. Histopathological Assessment

Four weeks post-administration, the whole brains were harvested. Brain tissue sections were subjected to Nissl and Masson’s trichrome staining. To assess the therapeutic efficacy of GNT-NS, histopathological changes in the hippocampus were evaluated under a microscope by quantifying the neuronal population and neurofibrillary tangle pathology. Representative images were obtained from the predefined CA1 region of the hippocampus using consistent microscope settings within the same staining experiment. For each animal, three non-overlapping brain sections at comparable anatomical levels were selected for quantitative analysis. Image quantification was performed using ImageJ software (version 1.54g; National Institutes of Health, Bethesda, MD, USA). Nissl-stained sections were used to quantify neuronal populations in the hippocampal region, whereas Masson’s trichrome-stained sections were used to evaluate neurofibrillary tangle-related pathological changes.

### 2.9. In Vitro Safety Evaluation of GNT-NS

A549 and RPMI 2650 cells were cultured in DMEM and MEM, respectively. Both media were supplemented with 10% fetal bovine serum (FBS) and 1% penicillin-streptomycin (100 U/mL), with 1% L-glutamine additionally added to the MEM. The cells were incubated at 37 °C in a 5% CO_2_ environment. Cells in the logarithmic growth phase were seeded into 96-well plates at a density of 2 × 10^4^ cells/mL (100 μL/well) and incubated for 24 h. Subsequently, the supernatant was removed, and the cells were treated with 100 μL of fresh medium containing either GNT-NS or physiological saline (control) for an additional 6 h. Cell viability was then assessed using a CCK-8 assay following the manufacturer’s instructions, with absorbance measured at 450 nm using a microplate reader.

### 2.10. Statistical Analysis

All quantitative data are presented as mean ± standard deviation (SD). Statistical analyses were performed using GraphPad Prism software (version 10.4.1; GraphPad Software, San Diego, CA, USA). Normality and homogeneity of variance were assessed before parametric analyses using GraphPad Prism. When these assumptions were not met, appropriate non-parametric tests were considered. For comparisons among multiple groups with one independent variable, one-way analysis of variance (ANOVA) followed by Tukey’s post hoc test was used. For datasets involving two factors, two-way ANOVA followed by Tukey’s post hoc test was used. For repeated measurements from the same animals, two-way repeated-measures ANOVA was applied where appropriate. Nonlinear regression analysis was used for curve fitting where appropriate. A value of *p* < 0.05 was considered statistically significant. Statistical significance was indicated as * *p* < 0.05, ** *p* < 0.01, *** *p* < 0.001, and **** *p* < 0.0001.

## 3. Results and Discussion

### 3.1. Characterization

**Figure 2:**
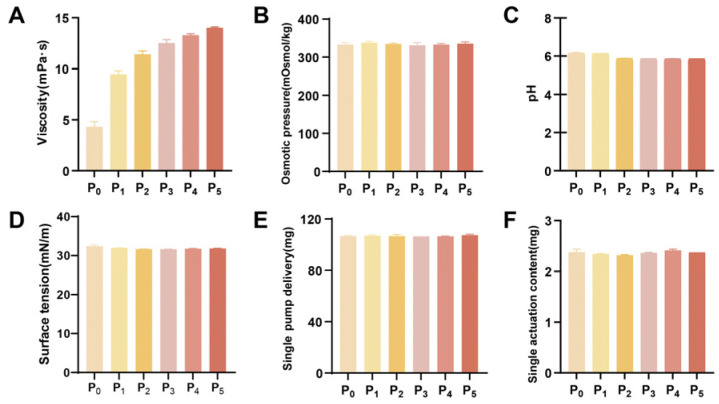
Characterization of GNT-NS. The viscosity (**A**); osmolality (**B**); pH of GNT-NS (**C**); Surface tension (**D**); Single pump delivery (**E**) and single actuation content (**F**) (*n* = 3).

The physicochemical properties of the GNT-NS formulations with varying PVP concentrations (P_0_–P_5_) are evaluated in Figure 2. As expected, viscosity increased proportionally with PVP concentration (Figure 2A). Crucially, this modification did not significantly alter the other fundamental properties or drug delivery performance. The osmotic pressures (280–380 mOsmol/kg, Figure 2B) and pH values (~6.0, Figure 2C) were unaffected by PVP, which is essential for maintaining physiological compatibility, preventing nasal mucosal irritation, and ensuring patient compliance. Furthermore, the surface tension of all solutions was maintained at approximately 32.00 mN/m (Figure 2D), while the single pump delivery dose and single actuation content remained stable at 106.00 mg and >2.3 mg, respectively (Figure 2E,F). The stability of these parameters successfully isolates viscosity as the sole independent variable, thereby establishing a rigorous scientific foundation for evaluating its specific impact on subsequent spray performance. Overall, despite the modulation in viscosity, all formulations (P0–P5) provide an optimal nasal cavity environment and are consistent with the relevant US FDA standards for nasal sprays [29,30].

### 3.2. Influence of Formulation Viscosity on Droplet Size Distribution

**Figure 3:**
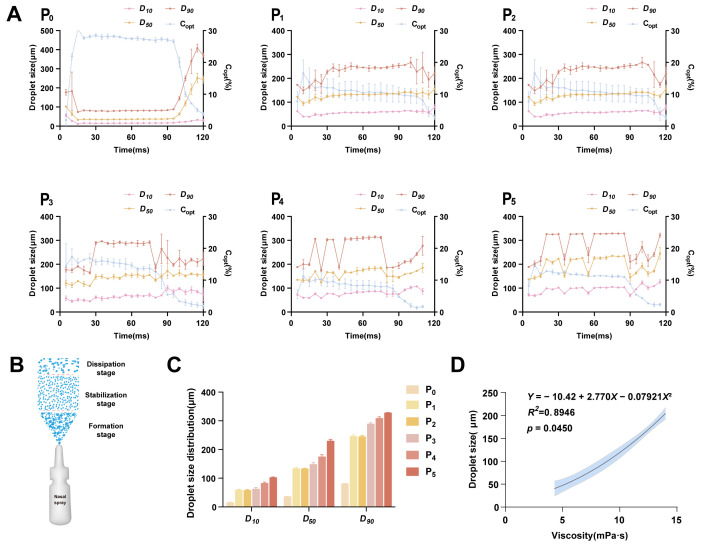
Droplet size distribution of GNT-NS. Profiles of droplet size and *C*_opt_ during the spray process (**A**). Schematic diagram of the formation, stabilization, and dissipation stages (**B**). Droplet size distribution during the stabilization stage (**C**). Correlation between droplet size and viscosity (**D**). The solid line represents the fitted second-order polynomial regression model, and the shaded area represents the 95% confidence interval of the fitted curve. *R*^2^ and *p* values are indicated in the plot (*n* = 3).

To investigate the influence of viscosity on the spray process, the droplet size of GNT-NS was monitored. The spray plume consists of numerous droplets, whose size and distribution significantly influence intranasal deposition patterns. The optical concentration (*C*_opt_), *D*_10_, *D*_50_, and *D*_90_ of formulations with varying viscosities were monitored in real time at 5 ms intervals throughout the spraying process. As illustrated in Figure 3A, *C*_opt_ exhibited a sharp initial increase, followed by a stable plateau, and ultimately a rapid decline. Accordingly, the spray process was divided into formation, stabilization, and dissipation phases (Figure 3B). During the stabilization phase, droplet size increased with formulation viscosity (Figure 3C), exhibiting a strong positive correlation with *D*_50_ (*R*^2^ = 0.8946) (Figure 3D). This can be attributed to increased internal cohesive forces at higher viscosities, which impedes liquid atomization and facilitates the formation of larger droplets [31,32]. This correlation suggests that droplet size can be adjusted by modulating formulation viscosity. Optimal droplet sizes should range from 10 to 300 μm to prevent pulmonary inhalation associated with extremely fine particles [33] and premature deposition in the nasal vestibule or anterior leakage caused by oversized droplets [31,34]. The droplet sizes of formulations P_1_–P_3_ fell within this target range, suggesting that viscosity modulation can adjust droplet size to a range suitable for nasal deposition.

### 3.3. Influence of Formulation Viscosity on Plume Geometry and Spray Pattern

**Figure 4:**
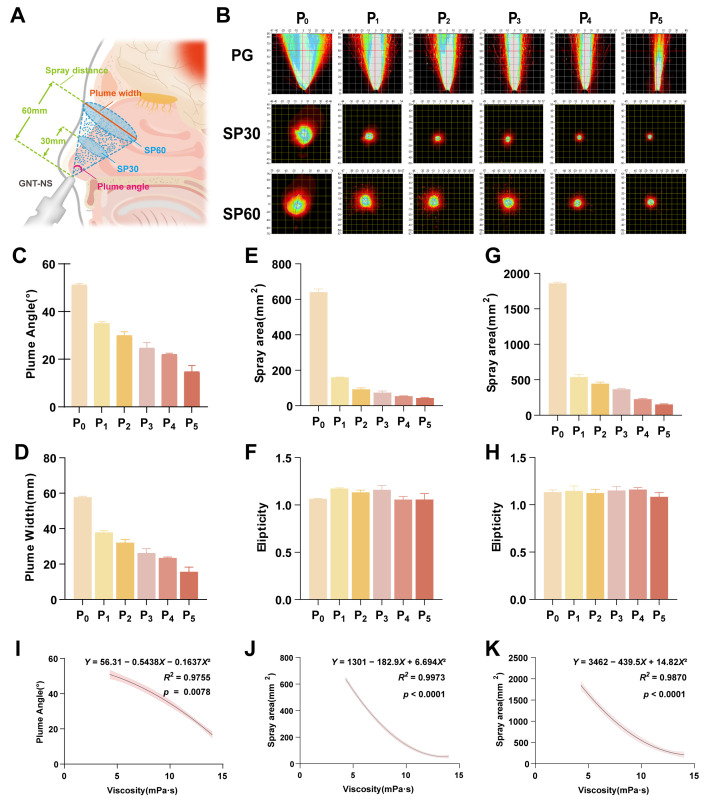
Plume geometry and spray pattern evaluation of GNT-NS. Schematic of plume geometry and spray pattern (**A**). Images of PG, SP30, and SP60 (**B**). Plume angle (**C**) and width (**D**). SP30 (**E**) and its ellipticity (**F**). SP60 (**G**) and its ellipticity (**H**). Correlations between viscosity and plume angle (**I**), SP30 (**J**) and SP60 (**K**). The solid lines represent fitted second-order polynomial regression models, and the shaded areas represent the 95% confidence intervals of the fitted curves. *R*^2^ and *p* values are indicated in the plots (*n* = 3).

Characterizing the nasal spray plume is essential for understanding how drug deposition in the nasal cavity during product development [35]. The International Pharmaceutical Aerosol Consortium on Regulation and Science (IPAC-RS) has identified plume geometry (PG, including plume angle and plume width) and spray pattern (SP, including spray area and ellipticity) as important performance metrics. Figure 4A shows the schematic of the plume angle, plume width, and spray areas at distances of 30 mm and 60 mm (SP30 and SP60), which could be measured using a high-speed camera-based droplet measurement system. Figure 4B illustrates the spray process of the GNT-NS formulation, where PG represents the emitted conical structure, and SP30 and SP60 correspond to the spray areas when the droplets reach the nasal vestibule and respiratory regions, respectively. The results indicate that as viscosity increases, the spray plume angle decreases from 51.20° to 14.80°, and the plume width narrows from 57.68 mm to 15.64 mm (Figure 4C,D). The narrower plume generated by PVP-containing formulations may reduce anterior nasal deposition and favor delivery to deeper nasal regions. However, excessive viscosity also leads to oversized droplets, causing premature gravitational deposition in the anterior cavity and compromising olfactory delivery efficiency. Since a plume angle of less than 45° is essential for navigating nasal passages and reaching the olfactory area, formulations P_1_~P_5_ are viable candidates for nose-to-brain delivery [36].

During nasal spray administration, droplets are initially intercepted by the nasal vestibule, making SP30 a critical parameter for effective drug delivery. As shown in Figure 4E, the rational tuning of viscosity significantly reduced the SP30, facilitating droplet passage through the nasal vestibule. Furthermore, since the minimum cross-sectional area of a typical adult nasal cavity is <400 mm^2^ [37], sprays with SP60 values below this threshold are more likely to pass through the nasal valve and reach the respiratory and olfactory regions. Peripheral droplets exceeding this threshold are retained in the nasal vestibule, resulting in drug loss. Notably, formulations P_1_–P_3_ exhibited optimal lower SP60 values (Figure 4G), making them particularly favorable for deep olfactory delivery. This parameter was therefore considered relevant to subsequent regional deposition analysis. Regarding spray geometry, ellipticity values close to 1 indicate an ideal circular pattern, whereas elevated ellipticity causes deviation from the plume center, leading to premature deposition and impeded deep nasal delivery [38]. All formulations exhibited ellipticity values close to 1 (<1.2), indicating a uniform and stable spray distribution pattern (Figure 4F,H). Finally, significant negative correlations were observed between viscosity and both the plume angle (*R*^2^ = 0.9755) and spray area (SP30: *R*^2^ = 0.9973; SP60: *R*^2^ = 0.9870), confirming that viscosity modulation is an effective approach to optimize nasal spray performance (Figure 4I–K).

### 3.4. In Vitro Nasal Distribution and Olfactory-Region Deposition of GNT-NS

**Figure 5:**
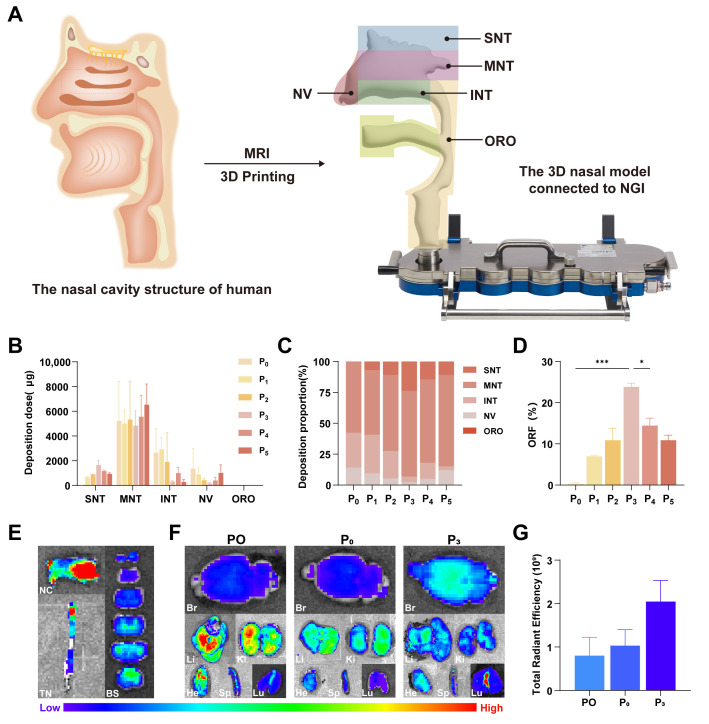
In vitro nasal distribution and fluorescence imaging of GNT-NS. Modular 3D-printed nasal cast coupled with NGI (**A**); Nasal deposition distribution dose (**B**) and proportion (**C**) of GNT-NS; ORF of GNT-NS (**D**); The nose-to-brain delivery process of GNT-NS (NC: Nasal cavity; TN: Trigeminal nerve; BS: brain slicing) (**E**); Total radiant efficiency among P_0_, P_3_, and PO groups (Br: Brian; Li: Liver; Ki: kidney; He: Heart; Sp: Spleen; Lu: Lung) (**F**); Semi-quantitative analysis of the whole brain (**G**) (*n* = 3, * p < 0.05 and *** p < 0.001).

To investigate the effect of formulation viscosity on drug distribution patterns and targeting efficiency to the olfactory region, we utilized a 3D-printed human nasal cavity model, which was reverse-engineered from authentic clinical magnetic resonance imaging (MRI) data, integrated with a next generation impactor (NGI) (Figure 5A). The NV region corresponded to the nasal vestibule, an area characterized by low blood flow, a thick cellular barrier, and limited surface area, rendering it unsuitable for drug absorption. While the INT and MNT (representing the respiratory zone) are more conducive to absorption, their extensive mucosal capillary networks lead to systemic circulation, which limits direct brain delivery [39]. Conversely, the SNT corresponds to the olfactory zone, where deposited drugs can bypass systemic circulation and directly enter the brain via olfactory nerves [12,15].

Figure 5B,C presents the deposition doses and proportions across these anatomical sites. The results indicate that most droplets were deposited in the nasal cavity, with minimal deposition in the oropharynx (ORO) region. This suggests that the aerosols largely avoided the lower respiratory tract, ensuring administration safety and preventing potential pulmonary toxicity. The proportion of drugs deposited in the SNT region was defined as the Olfactory Region Fraction (ORF). As shown in Figure 5D, the ORF for each formulation initially increases and then decreases with increasing viscosity. Notably, the ORF for formulation P3 reached 23.85%, representing a 74.53-fold increase compared to the unregulated formulation P_0_. In terms of absolute deposition, the amount of drug deposited in the olfactory region increased from 28.51 ± 20.70 μg for P_0_ to 1656.25 ± 378.28 μg for P_3_. This significant improvement is attributed to the optimal droplet size, spray geometry, and spray pattern of P_3_, which successfully minimized drug deposition in the NV and respiratory zones while maximizing targeted delivery to the SNT. Together, these results indicate that the optimized spray performance of P_3_ was associated with increased olfactory-region deposition. This finding provides in vitro support for the proposed relationship between formulation properties, spray performance, and olfactory-region deposition. However, because the nasal cast model was derived from a single adult volunteer, future studies can further evaluate how inter-individual nasal anatomical variability influences olfactory-region deposition.

### 3.5. In Vivo Biodistribution of Optimized GNT-NS

After intranasal administration of P_3_, drug fluorescence was detected in the nasal cavity, trigeminal nerve, olfactory bulb, and brain tissue, suggesting that GNT may be transported via trigeminal nerve to the olfactory bulb and subsequently distributed within the brain parenchyma (Figure 5E). Further semi-quantitative analysis showed that the total radiant efficiency in brain tissue was higher in the P_3_ group than in the P_0_ and commercial oral formulation (PO) groups (Figure 5F,G). At 2 h after administration, the whole-brain fluorescence signal in the P_3_ group was approximately 1.99-fold higher than that in the P_0_ group. These results indicate that optimization of ORF may increase brain-associated exposure after intranasal administration, providing supportive evidence for subsequent pharmacodynamic evaluation.

### 3.6. In Vivo Pharmacodynamic Studies on AD Rats and Safety Evaluation

#### 3.6.1. Learning and Memory Ability Assessments

**Figure 6:**
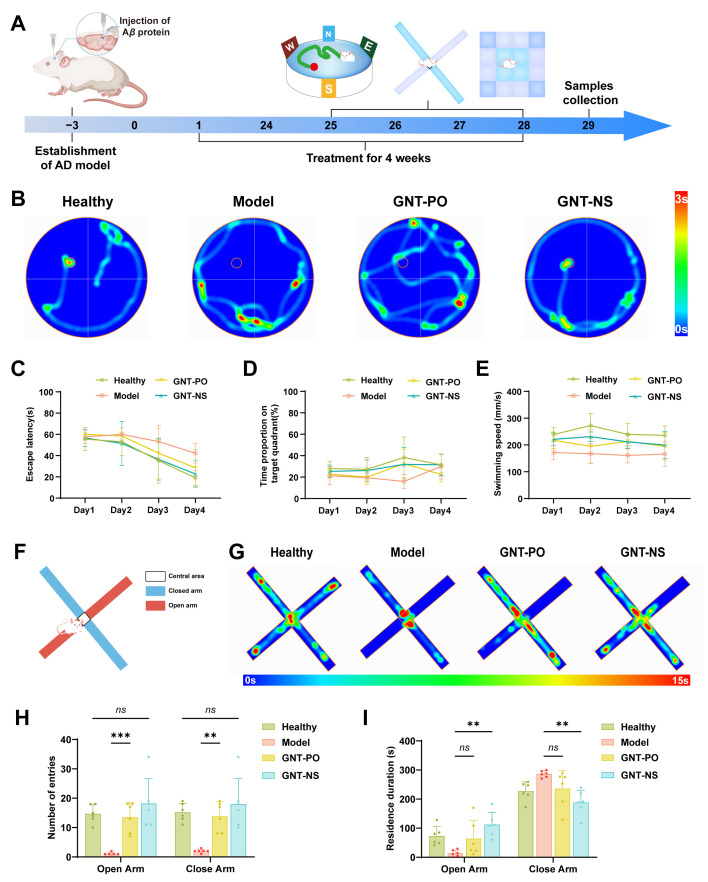
In vivo pharmacodynamic studies of GNT-NS on the AD model. Construction of AD models and experimental protocol (**A**). Motion trajectory heatmaps (**B**), with the red circles indicating the location of the hidden platform. Escape latency (**C**), the time proportion in the target quadrant (**D**), and swimming speed (**E**) of rats in the Morris water maze test. Illustration of the elevated plus maze (**F**). Motion trajectory heatmaps (**G**), number of entries (**H**), and residence duration (**I**) in the open and closed arm during the elevated plus maze test (*n* = 6). (** *p* < 0.01, *** *p* < 0.001; *ns*, not significant).

To evaluate the enhanced therapeutic efficacy of GNT-NS on AD, behavioral evaluations were conducted using AD rat models (Figure 6A). The Morris water maze test was employed to assess spatial learning and memory abilities. On the first day of the experiment, the swimming speeds of the rats in all groups were similar, indicating that A*β*_1–42_ administration did not impact their motor abilities. The motion trajectory heatmap (Figure 6B) revealed that while the model group displayed scattered and erratic movements indicative of significant impairments in spatial learning and memory, rats in the healthy and GNT-NS groups exhibited clear, directed navigation toward the hidden platform.

Quantitative assessments of learning and memory were based on three key metrics: escape latency, time proportion on target quadrant, and average swimming speed (Figure 6C–E). As training progressed, memory acquisition improved across treatment groups. Notably, the GNT-NS group exhibited the most significant improvement in escape latency, which was 21.50% and 11.66% shorter than those of the model and GNT-PO groups, respectively, reaching levels comparable to the healthy group. Similarly, GNT-NS treatment significantly prolonged the time proportion on target quadrant by 32.71% compared to the model group. Furthermore, the swimming speed of the GNT-NS group (215.80 mm/s) aligned with that of the healthy group, being 29.69% and 5.22% faster than the model and GNT-PO groups, respectively. Collectively, these results suggest that GNT-NS improved spatial learning acquisition and cognitive behavioral performance in AD rats. The greater behavioral improvement observed in the GNT-NS group is consistent with the enhanced olfactory deposition and brain-associated exposure observed in the deposition and biodistribution studies. These findings suggest that optimized formulation properties may contribute to improved pharmacodynamic outcomes.

#### 3.6.2. Exploration Behavior and Anxiety Assessments by the Elevated Plus Maze

Anxiety and exploratory behavior, common neuropsychiatric effects linked to AD, were assessed using the elevated plus maze (Figure 6F) [40]. This test evaluated these behaviors by recording the number of entries and the residence duration in the open arms (Figure 6G–I). The model group exhibited significantly decreased open-arm activity, with both the number of entries and duration being substantially lower than those of healthy rats. Treatment with GNT-NS and GNT-PO significantly reversed these deficits; notably, the residence duration in the open arms increased to 8.13 and 4.65 times that of the model group, respectively. Thus, GNT-NS improved anxiety-related and exploratory behaviors in AD rats and showed greater pharmacodynamic effects than the commercial oral formulation under the present dosing conditions. This improved pharmacodynamic performance supports the proposed optimization rationale linking formulation properties, spray performance, olfactory-region deposition, and therapeutic outcomes. By adjusting formulation physicochemical properties, GNT-NS improved spray performance and olfactory-region deposition, which may have contributed to the observed pharmacodynamic benefits in the AD rat model.

#### 3.6.3. Exploration Behavior and Anxiety Assessments

**Figure 7:**
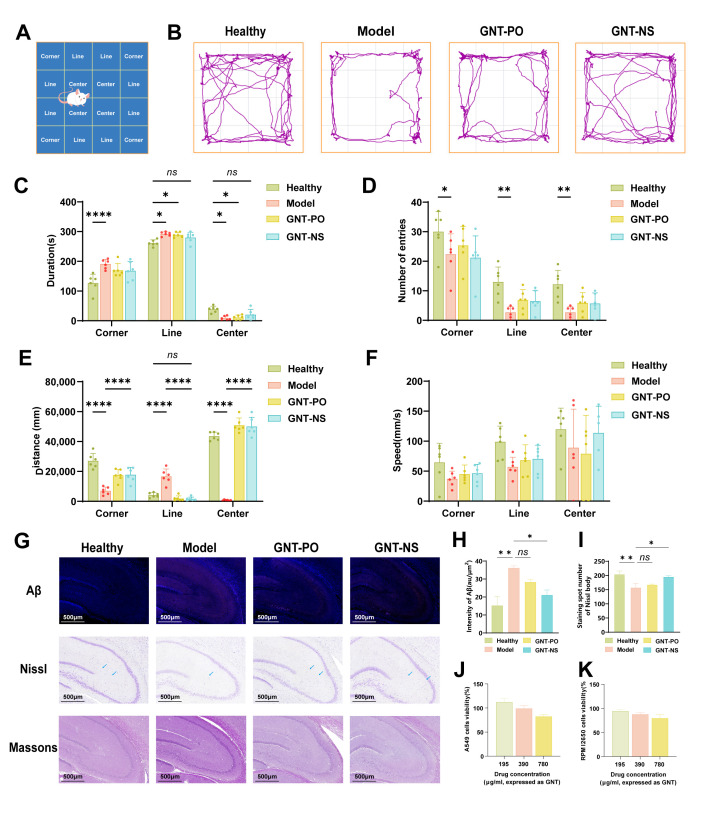
Behavior evaluation and histological assessment of the AD rat models. Illustration of the open field test (**A**). Motion trajectory heatmaps (**B**), duration (**C**), number of entries (**D**), total distance (**E**), and average speed (**F**) of rats in the corner, line, and center areas (*n* = 6). Sections stained with immunofluorescence, nissl, and masson trichrome staining (**G**); the blue arrows indicate representative Nissl-stained neuronal cell bodies. Fluorescence intensity of A*β* (**H**); Number of Nissl bodies (**I**); Cell survival rates of A549 (**J**) and RPMI 2650 cells (**K**) treated with different concentrations of GNT-NS (*n* = 3). (* *p* < 0.05, ** *p* < 0.01 and **** *p* < 0.0001; *ns*, not significant).

The open field test assessed the spontaneous behavior and activity levels of rats to evaluate the effects of various treatments on exploratory behaviors and anxiety (Figure 7A). In this assay, distance traveled, and average velocity served as indicators of motor activity, while the frequency of entries and duration in the central zone reflected exploratory drive and anxiety levels. The model group exhibited restricted motor paths primarily localized to the periphery of the enclosure, indicating significantly diminished central activity and elevated anxiety.

Following treatment, both the GNT-NS and GNT-PO groups showed significant improvements across all motor and exploratory metrics (Figure 7B–F). Notably, the GNT-NS group demonstrated the most effective recovery, restoring activity levels to those comparable with healthy rats. Specifically, the duration in the central zone for the GNT-NS group was 2.3 and 1.83 times longer than that of the model and GNT-PO groups, respectively. These findings indicate that GNT-NS produced more pronounced behavioral improvement than the commercial oral formulation under the present experimental conditions. Because both formulations were administered at the same galantamine-equivalent dose, the results support the pharmacodynamic advantage of the optimized nasal formulation under this dosing regimen.

#### 3.6.4. Histopathological Assessment and Safety Evaluation

To evaluate the neuroprotective effects of GNT-NS, histological assessments of the rat hippocampus were conducted using immunofluorescence staining for A*β* (Figure 7G). Fluorescence imaging revealed that the model group exhibited significant pathological A*β* deposition, which was markedly reduced following treatment. Fluorescence imaging showed pronounced A*β* deposition in the model group, which was reduced after treatment. Quantitative analysis further showed higher A*β* fluorescence intensity in the model group than in the treatment groups, while GNT-NS reduced this signal toward the level observed in healthy controls (Figure 7H).

To further evaluate neuronal integrity, Nissl staining was performed [41]. Quantification revealed that the GNT-NS group possessed 1.24 times more Nissl bodies than the model group, whereas the GNT-PO group exhibited only a marginal increase (Figure 7I). Additionally, Masson’s trichrome staining was utilized to detect neurofibrillary tangles (NFTs) in the hippocampus. The model group exhibited dense staining characteristic of abnormal protein aggregates. In contrast, both the healthy control and GNT-treated groups lacked prominent NFTs (Figure 7G). Collectively, these histological results indicate that disease progression leads to severe neuronal loss, which is effectively mitigated by GNT treatment. Compared with the commercial oral formulation, GNT-NS produced more pronounced histological improvement under the present experimental conditions, indicating the pharmacodynamic advantage of the optimized nasal formulation. These improvements were consistent with the enhanced the olfactory-region deposition and increased brain exposure observed for GNT-NS, suggesting that formulation optimization may have contributed to improved therapeutic outcomes by facilitating nose-to-brain delivery of GNT. Meanwhile, no obvious adverse signs were observed during the animal experiments. The cytotoxicity assays in A549 and RPMI 2650 cells also showed no marked inhibition of cell proliferation after exposure to GNT-NS, further supporting the preliminary biocompatibility of the formulation (Figure 7J,K). Nevertheless, additional studies using transgenic AD models and more comprehensive long-term safety evaluations are still needed to further validate the pharmacodynamic relevance and safety profile of GNT-NS under more disease-relevant pathological conditions.

## 4. Conclusions

In this study, we developed a galantamine nasal spray (GNT-NS) with enhanced olfactory-region deposition for nose-to-brain delivery in AD treatment. By systematically elucidating the cascade relationship among physicochemical properties, spray performance, and olfactory deposition, we demonstrated that rational modulation of formulation viscosity dictates plume geometry and droplet size distribution, facilitating deep nasal delivery to the olfactory region. Consequently, the optimized formulation P_3_ achieved an olfactory deposition fraction of 23.85% in a physiologically realistic 3D-printed human nasal cavity model. Biodistribution studies confirmed that this enhanced olfactory deposition translated into significantly increased brain drug exposure following intranasal administration. Furthermore, in vivo pharmacodynamic studies demonstrated that the optimized GNT-NS produced significant cognitive improvements in AD rat models, substantially outperforming commercial oral galantamine tablets. Ultimately, these findings suggest that rational optimization of nasal spray physicochemical properties can enhance olfactory-region deposition and brain drug exposure, thereby improving pharmacodynamic efficacy of galantamine for AD treatment via the nose-to-brain route. Before further clinical translation, future studies will systematically evaluate the long-term stability, nasal toxicity, pharmacokinetics, tissue distribution, device reproducibility, and human-model applicability of GNT-NS.

## Figures and Tables

**Table 1:** The formulations components of P_0_~P_5._

Formulation	GNT (%, *w*/*v*)	PVP (%, *w*/*v*)	EDTA-2Na (%, *w*/*v*)	Na2HPO4 (%, *w*/*v*)	DDM (%, *w*/*v*)	NaCl (%, *w*/*v*)	BAK (%, *w*/*v*)
P_0_	2.50	0.00	0.50	0.10	0.25	0.60	0.01
P_1_	2.50	1.00	0.50	0.10	0.25	0.60	0.01
P_2_	2.50	1.25	0.50	0.10	0.25	0.60	0.01
P_3_	2.50	1.50	0.50	0.10	0.25	0.60	0.01
P_4_	2.50	1.75	0.50	0.10	0.25	0.60	0.01
P_5_	2.50	2.00	0.50	0.10	0.25	0.60	0.01

## Data Availability

The data supporting the findings of this study are included in the article. Additional data are available from the corresponding author upon reasonable request.
